# Indents

**Published:** 1921-12

**Authors:** 


### INDENTS

**☞Brief Articles of Technical or Scientific Value will receive notice and comment. Contributions invited.**

---

Acid versus Alkaline Dentifrices. The effect of fruit juices in the mouth is now quite clearly understood. The common expression that a taste of orange or apple makes one's mouth water means that these mildly acidic fruit juices have the peculiar power to stimulate salivary flow.

More than that, it means that the saliva which responds to this stimulation is frequently more normal than was found in the same mouth during the pre-stimulated period. This is one of the chief reasons why fruit should form a part of each meal, why each meal should open and likewise close with fruit.

It produces a copious, fluid, alkaline saliva which is so essential in order that the oral cavity may function properly.

Latterly various investigators have found that dentifrices should be mildly acidic like fruit to assist nature in maintaining a normal saliva. They have, moreover, proven that alkaline mouth preparations are contra-indicated in the mouth and should be abandoned because they oppose nature in maintaining normal oral secretions.

---

Refitting Dentures. Recently there was demonstrated an apparently new method of making a loose plate fit, using wax of different melting points as the medium.

In February, 1887, Geo. R. H. Hutchinson wrote the following in the Items of Interest:

"I have been quite successful in using this method: Take a sheet of wax, warm it and place it evenly on the plate. Immerse in warm water till both are warm. Take the impression by letting the patient close the jaws firmly; remove, and trim off surplus wax on the edges, then flask in plaster. Heat, wash off all the wax and replace with rubber; close and vulcanize. For lower plates it is especially good.''

The principles employed in this method are the same, whether the water and the wax are heated with an electric water heater or some other method employed for this purpose 34 years ago.

The wax will compress the softer areas in the mouth and relieve the harder areas. There is no question that this method is of great help and offers many advantages if you are not familiar with the more scientific and exacting methods in the construction and adaptation of artificial dentures.—*Northwestern Journal.*

---

IMMEDIATE EXTRACTION AND DENTURE INSERTION. I believe that the best results in denture work are obtained where the patient is not allowed to go edentulous for a single day. I get the best results where the buccal and labial process is resected at time of extraction, gum trimmed to straight clean lines and sutured. Impression taken immediately and cast poured with plaster with setting hastened by potassium sulphate. This may be separated in five minutes and a base plate adapted to the cast of Graft's base plate material. The six anterior teeth are fused to the base plate. Ridges are built up in the posterior region to establish occlusion with the jaws at normal rest. These base plates are worn when the patient leaves the office, maintaining the normal relationship of the jaws, preventing the sagging of the muscles and strain and change in the mandibular joint. With this temporary base plate the patient can go on about his business unembarrassed and suffering much less pain and discomfort—the base plate acting as a splint or bandage protecting the wound in the mouth from irritation from air and tongue and food pressure. These base plates may be worn indefinitely, from one to several weeks to meet the pleasure and convenience of patient and dentist.—V. C. Smedley.

DON'T EXTRACT WITHOUT REASON. I don't think we should extract teeth that can be saved, or unless we have a very good reason for doing so. Personally, I will confess to having reached the point, after investigation and study—I have been doing X-ray work for many years—that whenever a focus exists which involves a considerable area at the apex, I advise the removal of that tooth and the thorough curetting of the socket. It must not be forgotten that the history of the patient's physical condition must be taken into consideration.

I used dichloramin-T for some time before Dr. Prinz brought it out in dental literature. He was kind enough to take me into his laboratory and explain its use to me. I have used it, and although in a number of cases there has been regeneration of bone, in others there was no beneficial response; but even in many of the former cases, upon checking it up with the X-ray later on, I found rarefaction again developing.

Please remember that in a pulpless tooth we have nothing left alive of that tooth but the cementoblasts that are in the cementum. Every odontoblast has died, and with it its fiber. The tooth proper is practically dead. This dead animal matter forms a pabulum for the microorganisms. We have been shown that teeth not only have one canal entering at the apex, but many of them; in fact, I think the majority have two or three, and in one instance of a molar exhibited by Dr. Grove, of Minneapolis, there were eight radiating from the main canal. Dr. Peters may fill one root canal, but besides that one there may be two others radiating from it, small possibly, but for a micrococcus they are full of pabulum. Bacteria will live at these openings on the sides of the roots. The Streptococcus viridans does not produce pus in pure culture. There is no pus present where there is only viridans infection, at the start at least. The Streptococci viridans become acclimated to their new environment, and securing their nutriment from the serum, eventually become aggressive and dangerous.

Dr. Cotton recently said: "There were 2,800 insane people in my institution. Five years ago many of them were sane. Last year we sent 70 per cent. of them home as cured by removing foci of infection from their teeth, tonsils or intestines."

The Bureau of Vital Statistics showed that in 1916 more people died of heart disease than of tuberculosis. Many of these people probably were free from infection five years previous to that time. Can you say definitely when these pulpless and infected teeth will cause trouble? I do not think you can. When a person is suffering from a secondary infection, and symptoms of toxemia from bacteria develop, the physician naturally asks, "Teeth, tonsils, or intestinal tract?" If of the modern school, he will advise, first of all, a careful X-ray examination of the mouth.

My experience with radiography has been a little different. I have yet to find a rarefied area at the apex of a vital tooth. I have opened such teeth which were under suspicion and secured a slight sensation but found the pulp in a disorganized condition, and dissolution slowly but surely taking place.

One picture of the apex of a pulpless tooth may show no rarefaction, yet another picture taken at a different angle may disclose such absorption. If a person over forty-five years of age, who is suffering from some obscure systemic condition, is referred to you by a physician, and the X-ray discloses pulpless teeth, some with active infection and others seemingly quiescent, I believe such patients should have all pulpless teeth removed. Like many others I have, for my own satisfaction, tested the apices of pulpless teeth which showed no rarefection by the X-ray, on blood agar, and secured a culture of streptococcus viridans.

In my opinion, the experience of men like Billings, Rosenow, Hartzell and others should guide us, and where a patient shows himself susceptible to these foci of dental infections, these areas should be eradicated and surgically cleaned up. It takes considerable courage to advise your patients to have seemingly good teeth extracted.--A. C. Fones, in *Cosmos*, June, 1921, Page 637.

---

SURGEON'S SOAP. I beg to present a formula for making a soap that I have been using in my practice for a good many years, one that thoroughly cleanses and does not injure the skin. It may also be used for washing patients' faces before operating.

Green soap, 35 oz.
Alcohol, 95 per cent., 9 oz.
Glycerin, 13 oz.
Salicylic acid, 94 gr.
Water, 90 oz.

If unable to obtain pure alcohol, that which contains a small amount of lysol can be used, but do not use alcohol containing formaldehyd or bichlorid of mercury.

Mix the soap with glycerin and salicylic acid and stir in a small amount of alcohol, constantly stirring until well mixed, then slowly pour in the boiling water, constantly stirring. After all the water is added, then add and stir in the remaining part of the alcohol. Let stand until cold, and filter through filter paper.—J. M. Rosenthal, in *Cosmos*, June.

---

PACKING VULCANITE. In calling attention to flasking and packing, I hardly think I can make a suggestion that has not been repeatedly dwelled upon, but at the same time it is apparently daily overlooked. As a rule the operator after approving his try-in-denture sends it to his own laboratory, or to the public laboratory with instructions to duplicate in vulcanite. No matter how well experienced a packer may be he cannot exactly estimate the amount of rubber. There is more on right than on left and vice versa. Any of us with a moment's thought can readily see how this effects the articulation as well as the adaption of the denture. The bolt flask is most universally used, and I believe in tightening of these bolts without due precaution often causes unsatisfactory results. The bolt flask can be successfully used, but a great deal of care must be used in the packing, and in securing approximal contact of the flask, the edges of the flask should be free of rust, ground and polished, and in securing approximal contact a vise should be used which will apply pressure in center of flask. The spring flask is more desired, it being easier to manipulate, giving an even pressure on all sides, and if the case is properly packed, with small vents for excess, a duplicate of the try-in-denture is almost assured.—Dr. W. H. Hoyt, in *Dental Facts.*

---

Oral Hygiene and Preventive Dentistry. If the dentists of America will join hands and start a country-wide movement to prevent cavities forming upon the occlusal surfaces of permanent molars they will do more for preventive medicine than all the root canal work ever invented or any other kind of dental reparative work.

The technic is simple. First, with a fine-pointed explorer loosen up any debris in the fissures. Place two or three drops of peroxide on the surface and work explorer in every part. Wash off with warm water. Dry and repeat with explorer and H2 O2. Wash off again. Place cotton rolls on either side of the tooth. Dry, then wash off with Daken solution. Dry. Now use Howe's method of silver nitrate and dry quickly so as not to allow too much discoloration. Dry once more. Mix S. S. White Silver Cement to the consistency of condensed milk. Place cement on tooth and with fine explorer work down into crevices. Place a small square of tinfoil, gauge sixty, upon the cement. Use a burnisher and burnish the tinfoil to the tooth in every direction, working from the center toward the edges. As soon as cement is set, peel off the tinfoil and any surplus can now be removed. This method will preserve the occlusal planes and all masticating efficiency.—Dr. T. B. Hyatt.

---

To Grasp Broaches in the Fingers. Warm the butt end of a nerve broach and smear this end of the broach with a little piece of hot gum shellac. When the shellac is cool enough place the thumb and first finger on the shellacked broach and roll into a pear-shaped ball of proper proportions and form to make the handle for holding the broach definitely. This makes a broach holder unnecessary, and it can be handled more delicately and safely.—T. C. Williams.

---

Pink Rubber Inset. Cut out the dark rubber spot of the form and size, and make a pink rubber inset from a piece of old pink rubber plate. Place the pink inset into place and apply a warm spatula and force the insert to place and hold until cold. Use as hot a spatula as practicable without burning the inset or plate. When cold finish and polish, and the patched inset will not be noticable.—G. C. Barkley.

---

An Important Decision. The Supreme Court of Massachusetts has just given a decision concerning a physician of that state which is worth noting. Dr. A. A. Lawrence, of Boston, is said to have committed an act which displeased the Board of Registration in Medicine, and the members of the board asked him to appear before them and explain the matter. This he refused to do, and appealed to the court to prevent the board taking any action in the matter, as he said he had been practicing medicine for years before the board was created and so had acquired certain vested rights, upon which the board had no right to infringe. The case of appeal came before the

Supreme Court, and that court ruled that while the right of a physician to toil in his profession is recognized and must be guarded against all unwarrantable interference, yet this right is not absolute and must yield to the paramount right of the government to protect the public health by rational means. The court said:—"A physician, however, skillful, who is guilty of deceit, malpractice or gross misconduct in the practice of his profession, even though not amounting to an offence against the criminal laws, well may be thought to be pernicious in relation to the health of the community. It is for the legislature to determine within reasonable limits in the exercise of the police power what the test shall be for moral character sufficient to enable one to continue in the practice of medicine. He has no vested right to prey upon society by the exercise of deceit, malpracticey or gross misconduct in the practice of his profession. His license to practice constitutes no contract of that nature." This assertion of the rights of society as overriding the rights of the individual is becoming increasingly common, and is well founded both in reason and in law.—*Oral Health.*

